# Regular exposure to a *Citrus*-based sensory functional food ingredient alleviates the BOLD brain responses to acute pharmacological stress in a pig model of psychosocial chronic stress

**DOI:** 10.1371/journal.pone.0243893

**Published:** 2020-12-28

**Authors:** Sophie Menneson, Yann Serrand, Regis Janvier, Virginie Noirot, Pierre Etienne, Nicolas Coquery, David Val-Laillet

**Affiliations:** 1 INRAE, INSERM, Univ Rennes, Nutrition Metabolisms and Cancer, NuMeCan, St Gilles, Rennes, France; 2 Phodé, Terssac, France; University of Ottawa, CANADA

## Abstract

Psychosocial chronic stress is a critical risk factor for the development of mood disorders. However, little is known about the consequences of acute stress in the context of chronic stress, and about the related brain responses. In the present study we examined the physio-behavioural effects of a supplementation with a sensory functional food ingredient (FI) containing *Citrus sinensis* extract (D11399, Phodé, France) in a pig psychosocial chronic stress model. Female pigs underwent a 5- to 6-week stress protocol while receiving daily the FI (FI, n = 10) or a placebo (Sham, n = 10). We performed pharmacological magnetic resonance imaging (phMRI) to study the brain responses to an acute stress (injection of Synacthen^®^, a synthetic ACTH-related agonist) and to the FI odour with or without previous chronic supplementation. The olfactory stimulation with the ingredient elicited higher brain responses in FI animals, demonstrating memory retrieval and habituation to the odour. Pharmacological stress with Synacthen injection resulted in an increased activity in several brain regions associated with arousal, associative learning (hippocampus) and cognition (cingulate cortex) in chronically stressed animals. This highlighted the specific impact of acute stress on the brain. These responses were alleviated in animals previously supplemented by the FI during the entire chronic stress exposure. As chronic stress establishes upon the accumulation of acute stress events, any attenuation of the brain responses to acute stress can be interpreted as a beneficial effect, suggesting that FI could be a viable treatment to help individuals coping with repeated stressful events and eventually to reduce chronic stress. This study provides additional evidence on the potential benefits of this FI, of which the long-term consequences in terms of behaviour and physiology need to be further investigated.

## Introduction

Psychosocial stress is of major concern in modern life conditions. Indeed, when repeated or prolonged, chronic stress is highly associated with the development of mood disorders (*i*.*e*. anxiety, depression) and other pathologies (*i*.*e*. metabolic, gastrointestinal, *etc*.) [1]. Mood disorders have been extensively studied in different rodent chronic stress models where the onset of depressive- and anxiety-like behaviours is described. They are also notably associated with an increased hypothalamo-pituitary-adrenal (HPA) axis secreting activity, monoamines imbalance, and a decreased hippocampal neurogenesis [2–5]. These alterations have also been observed in human patients [6–8]. There are evidences that people suffering from mood disorders might have difficulties to cope with new stressful situations [9, 10].

In human, brain reactivity to stress can be studied through *in vivo* brain imaging, by coupling with different clinical acute stress protocols. It is the case of the Montreal Imaging Stress Task (MIST), which is frequently used in laboratories to study psychosocial stress, and notably increases cortisolemia and modulates brain activity [11, 12]. Using pharmacological functional magnetic resonance imaging (phMRI), it is also possible to observe the effects induced by the direct injection of a drug of interest [13, 14], and this might be also used to investigate the effects of a pharmacologically-induced acute stress on brain responses. Pigs are considered as a good preclinical model for many research and biomedical applications. Their general anatomy, organs size ratio and physiology are comparable to those in humans. Particularly, their brain is also gyrencephalic, and most of the cerebral regions are comparable in terms of structure, vascularization, anatomy, growth and development [15, 16]. Moreover, digital stereotaxic atlases are available and many classical human imaging techniques have been implemented in the pig model [17, 18]. Thus, pig is a particularly relevant model to study brain responses using *in vivo* imaging [17, 19]. We have notably been working on the development of adequate techniques to discriminate the brain responses to gustatory [20] and olfactory stimulations [21] through fMRI in this model [17, 19]. The pig might thus be an adequate model to study the brain responses to a pharmacologically-induced acute stress, through pharmacological fMRI (phMRI).

The use of natural products such as plant extracts is gaining interest to prevent stress-related consequences. Numerous studies have investigated the effects of orange essential oils and *Citrus* extracts, under acute or chronic stress contexts, through environmental or oral exposure. Acute inhalation of sweet orange essential oils can reduce anxiety in humans [22, 23]. Acute anxiolytic-like and sedative effects have also been observed with inhalation of *Citrus sinensis* in rodents [24, 25]. Impacts of a prolonged exposure through inhalation to *Citrus* extracts have also been investigated in chronic stress models and showed anxiolytic- and antidepressant-like effects [26–28]. Several physiological parameters altered in chronic stress and mood disorders can also be regulated by *Citrus* extracts, such as HPA axis activity [28, 29], monoaminergic systems [26, 28], and neurogenesis [30, 31]. In previous studies using respectively fluoro-deoxy-glucose (FDG) positron emission tomography (PET) [32] and functional magnetic resonance imaging (fMRI) [21], we have shown that olfactogustatory and olfactory stimulation with a food ingredient mainly composed of *Citrus sinensis* extracts was notably able to modulate the reward and motivational brain circuit, that are usually impaired in chronic stress and depression resulting to anhedonia.

In this study, twenty female pigs were subjected to a psychosocial chronic stress described in [33] and received in parallel a sensory functional food ingredient (FI) mostly made of *Citrus sinensis* extract formulation, or a placebo. The effects of supplementation were assessed on depressive-like behaviour and circulating cortisol levels. After 5–6 weeks, we performed an fMRI session to investigate the impacts of a pharmacological acute stress on brain activity. The effect of previous chronic supplementation with the FI was also investigated on those brain responses. Our hypothesis was that, in a chronic stress context, a pharmacological stress might modulate the brain activity of regions associated with arousal and emotion regulation, and that a pre-supplementation with the FI might also modulate these responses.

## Materials and methods

### Animals and housing

Experiments were conducted in accordance with the current ethical standards of the European Community (Directive 2010/63/EU), Agreement No. C35-275-32 and Authorization No. 35–88. The Regional Ethics Committee in Animal Experiment of Brittany has validated the entire procedure described in this paper (project n° 2018020509294503).

Twenty Piétrain × (Large White/Landrace) female pigs born at the INRA experimental research station of Saint-Gilles (UEPR, France) were used in this study. Animal were subjected to a natural day/night cycle, with a light period progressively increasing from 13h30 to 15h30 along the experimental course. From 9 weeks of age, they were subjected to 5–6 weeks of multifactorial psychosocial stress as previously described in [33], consisting in the combination of social isolation, environmental impoverishment, and unpredictable stress consisting of sounds (sirens, metallic noises, gunshots, etc.) and lights randomly diffused (every 10 min +/- 30% during the day, every 120 min +/- 30% during the night). Animals were separated in two groups and received the sensory functional food ingredients D11399 (FI group, n = 10) or a control formulation D12250 (Sham group, n = 10), designed and manufactured by Phodé (Terssac, France).

### Functional ingredient

D11399 contained an active core (B524) mainly composed of *Citrus sinensis* extract (24–32%) and was formulated with excipients based on water and plant emulsifying agents (36–48%) to obtain a liquid water-dispersible galenic form. D12250 contained purified coco oil (24–32%) formulated with the same excipient as D11399. D11399 and D12250 were diluted at the rate of 2.1% with a blend of vegetal powder excipients (50% rice extract/50% maize extract). The powder products thus obtained were mixed in cottage cheese at the rate of 0.5%, and administrated once a day in the morning. Both groups received D11399 and D12250 at the dosage of 0.2 mg/kg live weight. The choice of the administration with cottage cheese came as a result from previous sensory analysis tests conducted in panels of 36 persons and investigating two potential ways of administration for further translation to human. Additional information about the diet and feed rations can be found in one of our previously published papers [33, 34].

### Study design

**Fig 1:**
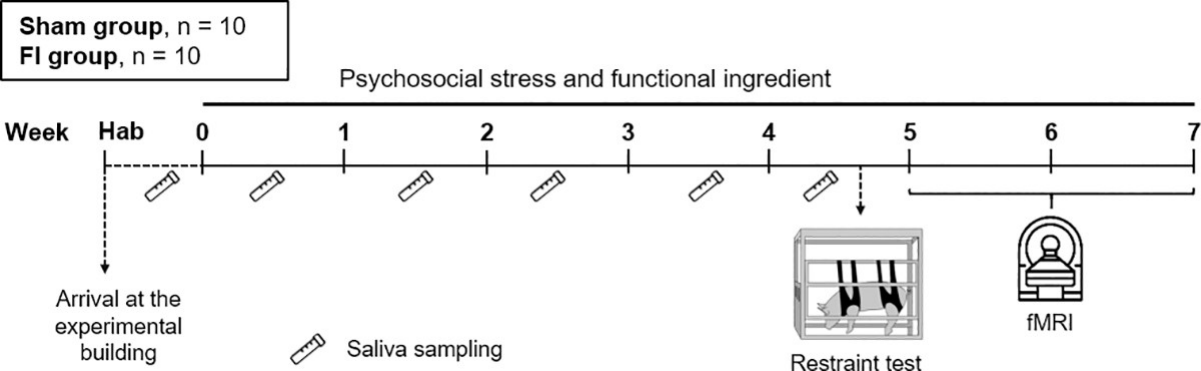
Experimental paradigm. At their arrival at the experimental building, pigs had one week (habituation, hab) to get used to their new environment and feed, and were then subjected to the psychosocial stress consisting in social isolation combined with environmental impoverishment and unpredictability, and to the functional ingredient. Depression-like behaviour was assessed in Week 5 in a restraint test. Saliva samples were collected 6 times, from the habituation week to Week 4. fMRI study was then conducted between Weeks 5 and 6 to investigate the effects of the supplementation with the functional food ingredient on brain responses to this ingredient and to an acute pharmacological stressor.

The entire experimental design is presented in Fig 1.

### Behavioural and physiological analyses

#### Restraint test

This test was adapted from rodents test [35] to study behavioural despair as a depression-like symptom in pigs [33] and performed after 4 weeks of stress and supplementation (Week 4). Pigs were equipped with two suspension harnesses and elevated with an electric-hydraulic system until the animals’ feet rose off the ground. The total duration of mobility, number of attempts to escape, and number of vocalizations were recorded for 5 min. A perseverance index was determined as the average duration of one escape attempt.

#### Salivary cortisol

Saliva samples were all collected once a week from the start of the experiment until 4 weeks of stress and supplementation (Week 4). At 8:30 a.m., overnight-fasted pigs were allowed to chew cotton buds (Salivette®, Sarstedt, Germany) for one minute. The buds were then rapidly centrifuged (2,500 g, 10 min, 4°C). Supernatants were stored at -20°C until cortisol quantification with a luminescence immunoassay kit (IBL, Hamburg, Germany).

#### Statistical analyses

Statistical analyses were performed using SPSS software version 25 *(IBM Corp*, *NY*, *USA)*. For the restraint test data, comparisons were made with a one-way ANOVA, and residuals were tested for normality with the Shapiro-Wilk test. For cortisol data, a repeated-measures ANOVA was performed to investigate the effect of time (time × group). Data are expressed as mean ± SEM. Differences were considered significant at *p* < 0.05.

### Functional imaging

#### Anaesthesia

Animals were subjected to *in vivo* brain imaging between 15 and 16 weeks of age, in Weeks 5–6. Initial sedation was performed with an intramuscular injection of ketamine (5 mg/kg–Imalgene 1000, Merial, Lyon, France) on overnight-fasted animals. Isoflurane inhalation (Aerane 100 ml, Baxter SAS, France) was used to suppress the pharyngotracheal reflex and then establish a surgical level of anaesthesia, 3–5% (less than 5 minutes) and 2.5–3% respectively. After intubation, anaesthesia was maintained with 2.5–3% isoflurane, and mechanical ventilation allowed adjustment of respiratory frequency at 17 breathing/minute with a tidal volume of 650 ml. The ventilation system was a homemade system consisting in a reanimation ventilator (Siemens SAL 900 D) coupled with an isoflurane reservoir [20, 21]. It worked as an open system, thus this 2.5–3% value is the one of the inhalation tank and not the one reaching the animal. Heart rate was always comprised between 80 and 150 beats per minute. Animals were covered with a blanket during imaging, the temperature was not recorded. The right ear was equipped with a venous route. Cotton wool with an additional headset were used to conceal the animal’s ears, and tape was used to maintain the eyes closed. Animals were euthanised at the end of the imaging session *via* an intravenous injection of T61 (1 ml/10kg) without awakening from anaesthesia.

#### Olfactory stimulation

We used a custom-made olfactory stimulation apparatus already used in previous studies [21], which was located outside the magnet-shielded room. Briefly, animals were equipped with a tube inserted in the right nostril, allowing air circulation into the entire nasal cavity. The odorant was formulated by Phodé (Terssac, France) and was composed of the active core B524 mainly based on *Citrus sinensis* extract (60–80%) (the same core as the functional food ingredient D11399), diluted in a vehicle (20–40%) composed of distilled water (60–80%) and glyceryl polyethylene glycol ricinoleate (20–40%). For olfactory stimulations, this formulation of odorant was diluted in distilled water (1 L) at the concentration of 0.105%. This odorant at this concentration had shown the highest level of brain responses, compared to another odorant and to the same odorant at a lower concentration in a previous study [21]. The control solution consisted of the vehicle diluted in distilled water at the concentration of 0.2%.

#### Stimulation paradigm

For each animal, a sequence of stimulation consisted in the alternation between odorant stimulation (FI: 30 sec, 4 L/min), and vehicle stimulation (Veh: 30 sec, 4 L/min), repeated 10 times. After a first MRI control acquisition (pre-Synacthen acquisition), animals were subjected to intravenous injection with 2.5 mg/kg of Synacthen^®^ and a second MRI acquisition (post-Synacthen acquisition) was performed 30 minutes after injection. Synacthen^®^ is an ACTH-related agonist, that pharmacologically mimics the effects of an acute stress through the activation of the HPA axis.

#### MRI image acquisition

Image acquisition was performed as previously described [20, 21, 36] on a 1.5-T magnet (Siemens Avanto) at the Rennes Platform for Multimodal Imaging and Spectroscopy (PRISM AgroScans, Rennes, France). Acquisitions were performed using a combination of coils (Body and Spine surface matrix coils, commercial products from Siemens, 6 channels were used for each) for optimized signal to noise ratio acquisition. Gradient shimming was performed automatically. *T1 weighted anatomical image acquisition*: a MP-RAGE sequence was adapted to the adult minipig anatomy (160 slices, 1.2x1.2x1.2 mm^3^, NA = 2, TR = 2400 ms, TE = 3.62 ms, TI = 854 ms, FA = 8°, acquisition duration 15 min). *BOLD (Blood-Oxygen-Level Dependent) signal acquisition*: an echo planar imaging sequence was adapted to pig head geometry (32 slices, TR/TE: 2500/40 ms, FA: 90°, voxel size: 2.5x2.5x2.5 mm^3^). The field of view was of 180 x 180 mm, the matrix size was 64^2^, and the total EPI imaging time was 10 min 30 (260 volumes x 2.5 seconds/volume, 4 initial volumes as dummy scans). For several animals, we detected a loss of MR signal in the frontal lobe due to the anatomical presence of an air cavity anterior to the brain. This type of artefact can be corrected [37, 38], but we decided to exclude this zone from the analysis which is depicted as dark regions on activation maps.

#### Data analysis and statistical image analysis

**Fig 2:**
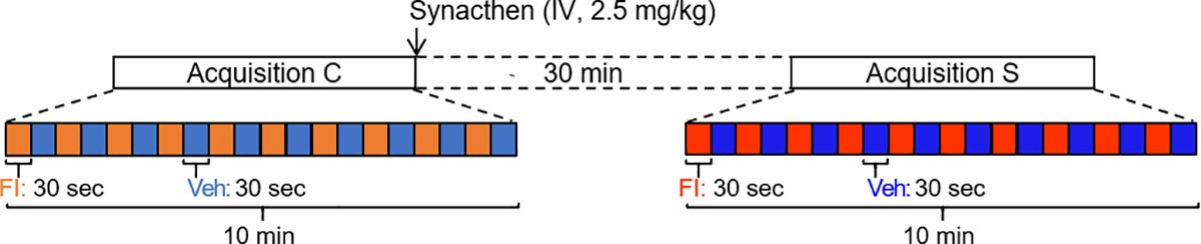
Stimulation and acquisition paradigm. For each animal, one acquisition was performed before the intravenous injection of Synacthen^®^ (pre-Synacthen acquisition) and one was made 30 minutes after (post-Synacthen acquisition). Each acquisition consisted in 10 alternations of a 30-sec odorant stimulation with the food ingredient and a 30-sec control stimulation with the vehicle. Contrasts were performed to investigate the brain responses to the food ingredient within the pre-Synacthen acquisition (Veh vs. FI) and the effects of Synacthen injection during vehicle stimulation between pre- and post-Synacthen acquisitions.

Data analysis was performed with SPM12 (version 6906, Wellcome Department of Cognitive Neurology, London, UK). After slice timing correction, realignment and spatial normalization on a pig brain atlas [39], images were smoothed with a Gaussian kernel of 4 mm. Due to limitations related to the size of the pig brain and the effect of anaesthesia on brain activity, we used a non-standard statistical analysis with regards to human statistical standards usually considering statistical significance at a cluster level with *p*-value < 0.05 under FDR correction. Further details regarding the validity and limitations of the statistical approach used in this model and paradigm are developed in [21]. *Voxel-based statistic*: first-level (within-individual contrast) and second-level (within-group contrast) statistics were assessed with a threshold set at p < 0.05 to produce the brain maps of activation. *SVC-based statistics (Small Volume Correction)*: twelve anatomical regions of interest (ROIs) corresponding to six bilateral brain structures previously studied in a chronic stress context [33] were used: hippocampus, amygdala, anterior and dorsolateral PFC, ventral and dorsal anterior cingulate cortex. They were studied with a *p*-value corrected with a Bonferroni correction at a threshold of 0.05 (peak level). The related uncorrected *p*-value threshold after Bonferroni correction was 0.0042. For voxel- and SVC-based statistics, no suprathreshold voxels were detected with false discovery rate-correction at *p* < 0.05. All the contrasts used in this study are presented in Fig 2 and emphasized in the panel A of each result (Figs 4A, 5A & 6A).

## Results

### The food ingredient did not affect the measured zootechnical, behavioural and physiological parameters

**Fig 3:**
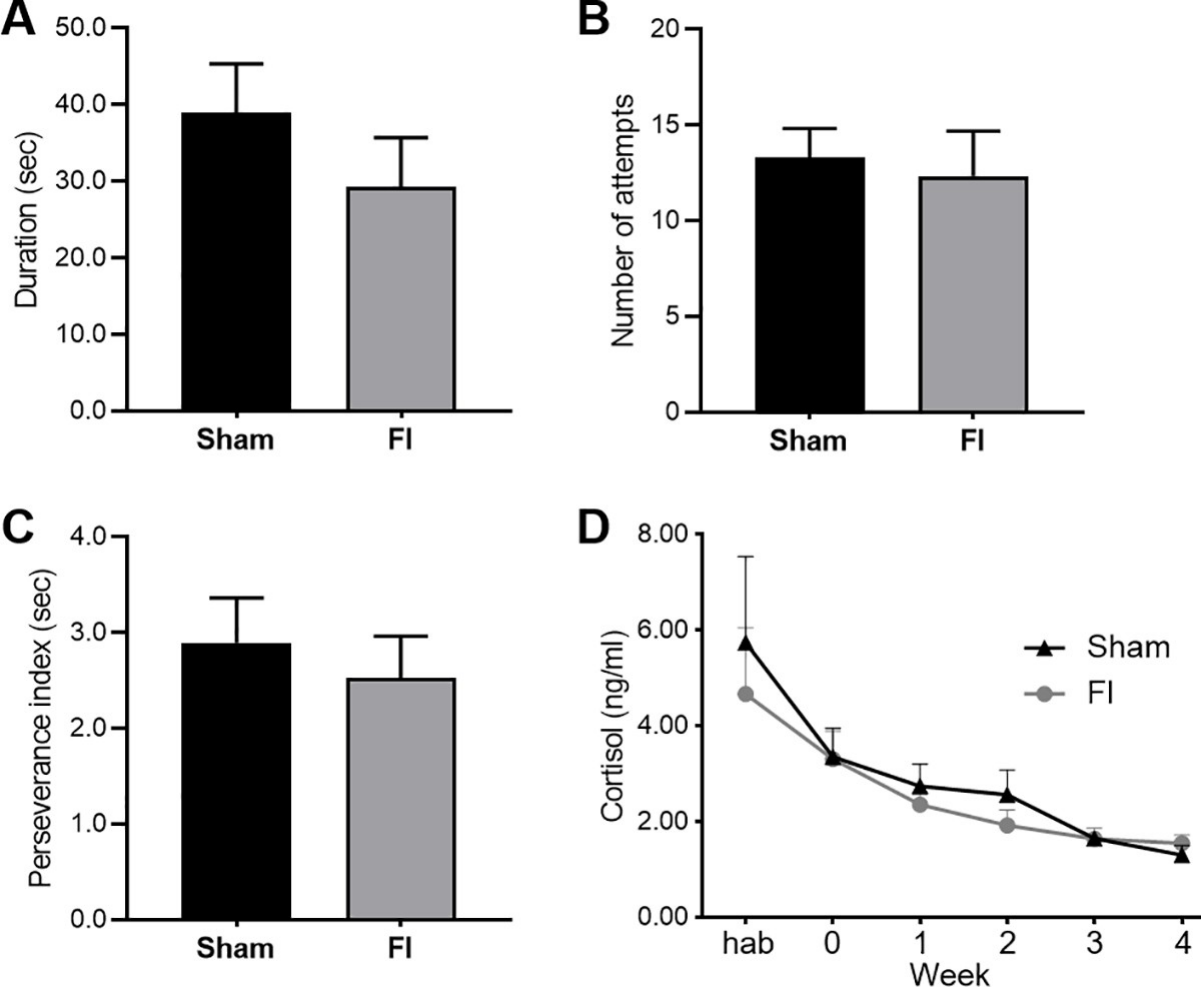
Behaviour and salivary cortisol evolution (SHAM, n = 10 and FI, n = 10). There was no difference of (A) duration of mobility, (B) number of attempts to escape, and (C) perseverance index between the control (Sham) and supplemented (FI) groups during the restraint test (animal number due to missing value: Sham, n = 10; FI, n = 9). The cortisol levels (D) did not differ between both groups along time (animal number due to missing value: Week hab: Sham, n = 8; FI, n = 8, Week 0: Sham, n = 9; FI, n = 10, Week 1: Sham, n = 10; FI, n = 10, Week 2: Sham, n = 10; FI, n = 10, Week 3: Sham, n = 9; FI, n = 10, Week 4: Sham, n = 10; FI, n = 10; mean +/- SEM, one-way ANOVA) although cortisol significantly decreased over time in both groups (p < 0.001, repeated-measures ANOVA).

Body weight did not differ between groups at the beginning of the study (Sham group: 25.7 ± 0.9 kg, FI group: 25.2 ± 1.2 kg, F(1,18) = 0.131, *p* = 0.72), and the supplementation did not induce growth difference (weight at the end of the study: Sham group: 58.9 ± 3.0 kg, FI group: 59.4 ± 2.6 kg, F(1,18) = 0.011, *p* = 0.92). Behavioural parameters observed in the restraint test did not differ: the duration of mobility (Sham group: 39.0 ± 6.4 sec, FI group: 29.3 ± 6.5 sec, F(1,17) = 1.134, *p* = 0.30), number of attempts to escape (Sham group: 13.3 ± 1.5, FI group: 12.3 ± 2.4, F(1,17) = 0.124, *p* = 0.73), number of vocalizations (Sham group: 153.9 ± 16.9, FI group: 145.3 ± 9.5, F(1,17) = 0.185, *p* = 0.67) and perseverance index (Sham group: 2.90 ± 0.47 sec, FI group: 2.53 ± 0.44 sec, F(1,17) = 0.328, *p* = 0.57). The salivary cortisol levels did not differ between groups at any moment of the experiment (at the start: Sham group: 5.74 ± 1.80 ng/ml, FI group: 4.66 ± 1.39 ng/ml, F(1,14) = 0., *p* = 0.64, and at the end: Sham group: 1.30 ± 0.20 ng/ml, FI group: 1.55 ± 0.17, F(1,18) = 0.876, *p* = 0.36), however it significantly decreased over time in both groups (F (5, 65) = 10,42, *p* < 0.0001) (Fig 3).

### Previous supplementation increased the brain responses to an olfactory stimulation with the food ingredient

**Fig 4:**
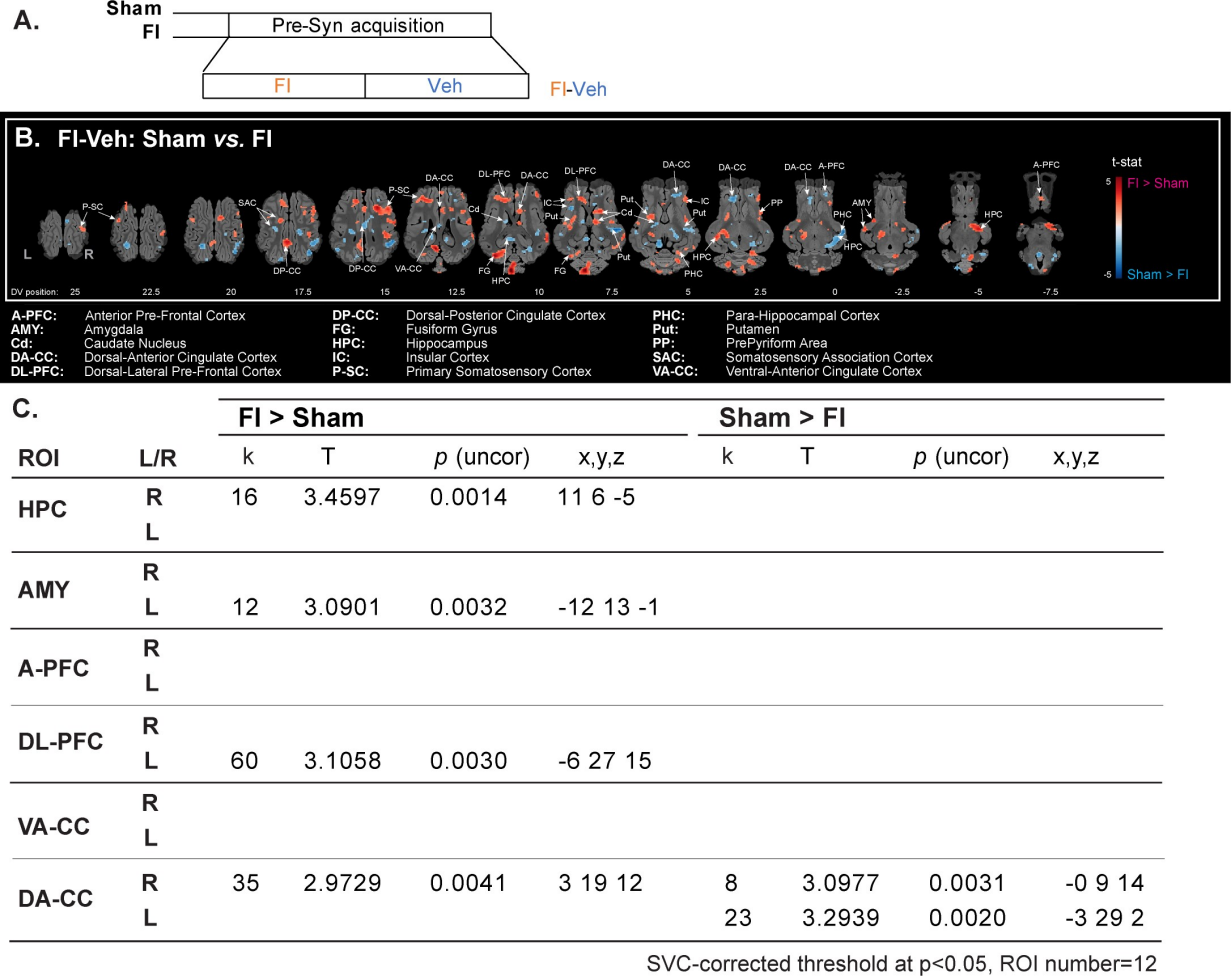
Previous supplementation increased the brain responses to an olfactory stimulation with the food ingredient. **(A)** Brain responses to the food ingredient were assessed in animals previously supplemented (FI, n = 10) or not (Sham, n = 10). FI: olfactory stimulation with the food ingredient, Veh: neutral stimulation with the vehicle. **(B)** Horizontal maps of brain BOLD responses to the food ingredient in both groups. p-value threshold = 0.05, k > 20, DV: dorsal-ventral position related to the posterior commissure (in mm). The part of the frontal cortices that was not covered with the average BOLD-based statistical maps is superimposed in dark grey on the anatomical maps. **(C)** SVC-based statistics: related regions of interest (ROIs) with uncorrected p-value that reached the criteria of p < 0.05 after Bonferroni correction.

The imaging contrast used to assess the effect of previous supplementation with the FI on responses to FI olfactory stimulation is described in Fig 4A. As seen on the brain activation maps (Fig 4B), the stimulation with the ingredient elicited higher brain responses in FI than Sham animals in the Primary Somatosensory Cortex (P-SC) and the Pre-Pyriform Area (PP), an olfactory relay/centre. FI animals also had higher brain responses in brain regions involved in associative learning and emotional processing, such as the Hippocampus (HPC), Parahippocampal Cortex (PHC) and Amygdala (AMY), as well as in the dorso-lateral Prefrontal Cortex (DL-PFC). Brain regions within the reward and motivational system were also more activated in FI animals, including the Caudate Nucleus (Cd) and Putamen (Put). Contrasted responses were observed in the cingulate cortex (CC; dorsal-posterior: DP-CC, dorsal-anterior: DA-CC and ventral-anterior: VA-CC).

#### Corrected SVC-based statistic (Fig 4C)

The responses to the olfactory stimulation with the ingredient were higher in the right HPC (*p* = 0.0014), left AMY (*p* = 0.0032), left DL-PFC (*p* = 0.0030) and right DA-CC (*p* = 0.0041) of FI animals. At the opposite, Sham animals showed higher bilateral brain responses in the DA-CC (right, *p* = 0.0031, left *p* = 0.0020).

### Pharmacological stress increased activity in several brain regions

**Fig 5:**
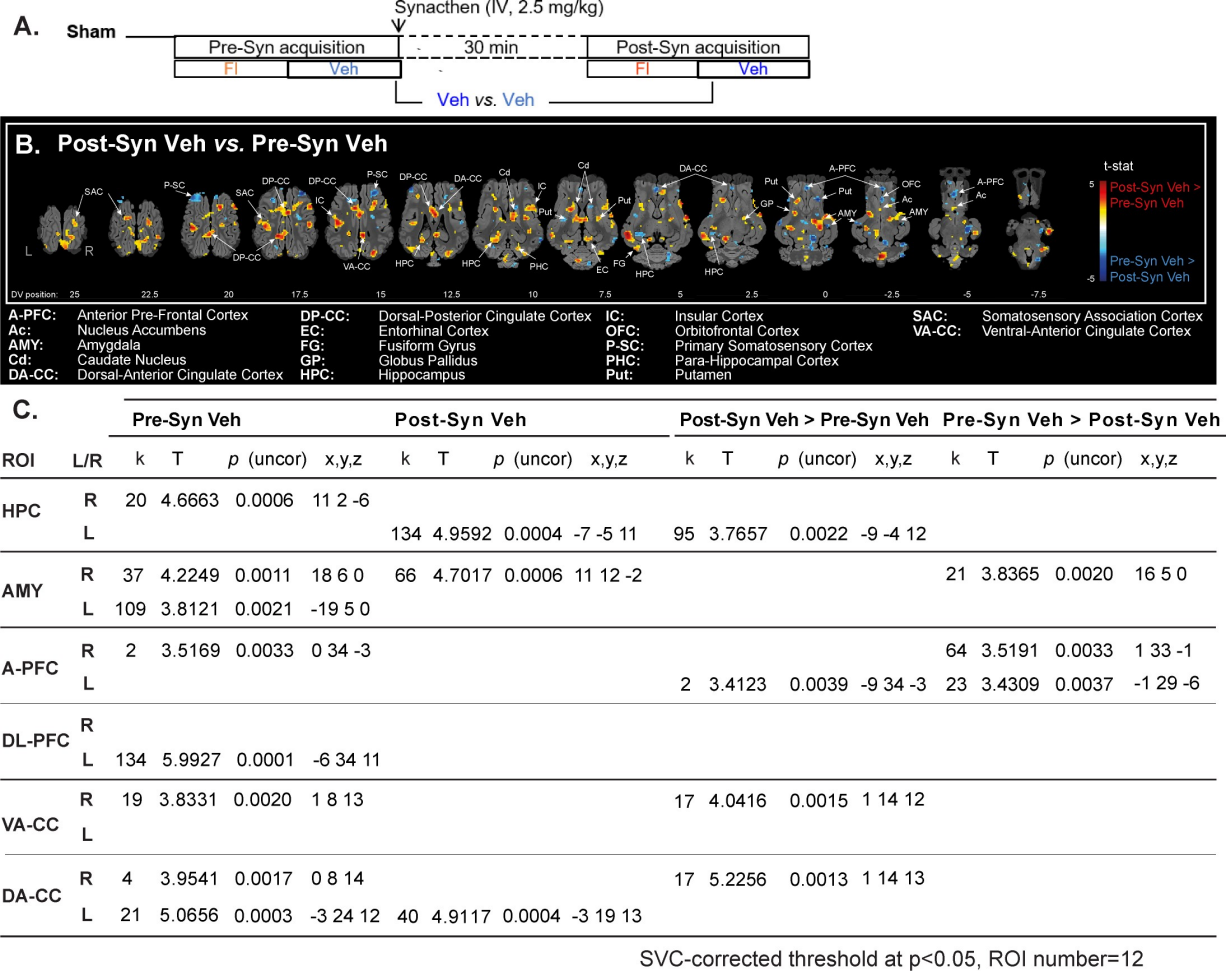
Pharmacological stress increased activity in several brain regions. **(A)** Brain activity during vehicle stimulation and responses to the injection of the pharmacological ACTH agonist (Synacthen®) in condition of vehicle stimulation were assessed in Sham animals (n = 10). FI: Olfactory stimulation with the food ingredient, Veh: Neutral stimulation with the vehicle. **(B)** Horizontal maps of brain BOLD responses to the injection. p-value threshold = 0.05, k > 20, DV: Dorsal-ventral position related to the posterior commissure (in mm). The part of the frontal cortices that was not covered with the average BOLD-based statistical maps is superimposed in dark grey on the anatomical maps. **(C)** SVC-based statistics: Related regions of interest (ROIs) with uncorrected p-value that reached the criteria of p < 0.05 after Bonferroni correction.

The imaging contrast used to assess the effect of pharmacological stress is presented in the Fig 5A. Overall, the brain activation maps (Fig 5B) show higher brain responses after the Synacthen^®^ injection under vehicle olfactory stimulation. Except in the P-SC, the pharmacological stress promoted a higher activity in brain regions associated with sensory perception, arousal and movement initiation, including the Somatosensory Association Cortex (SAC), Entorhinal Cortex (EC), Insular Cortex (IC), Fusiform Gyrus (FG) and Globus Pallidus (GP). Higher brain activity was also observed in brain regions involved in associative learning and emotions, including the ventral HPC, AMY, PHC, DA-CC, DP-CC and VA-CC. We could also detect decreased brain activation in the dorsal HPC and the right AMY. Reward and motivational regions such as the Cd, Put and nucleus accumbens (Ac) were also more activated after than before the injection. At the opposite, prefrontal regions associated with cognitive functions, decision-making and/or hedonic valuation, such as the anterior Prefrontal Cortex (A-PFC) and orbitofrontal cortex (OFC), had reduced brain activation after the pharmacological stress.

#### Corrected SVC-based statistic (Fig 5C)

Synacthen^®^ injection elicited an increased activation (Post-Syn Veh > Pre-Syn Veh) of the left HPC (*p* = 0.0022), left A-PFC (*p* = 0.0039), and right VA-CC (*p* = 0.0015) and DA-CC (*p* = 0.0013) under vehicle olfactory stimulation. At the opposite, the activity in the right AMY (*p* = 0.0020) and bilaterally in the A-PFC (right, *p* = 0.0033 and left, *p* = 0.0037) was reduced (Pre-Syn Veh < Post-Syn Veh).

### Supplementation with the food ingredient attenuated the global increased brain activity elicited by the pharmacological stress

**Fig 6:**
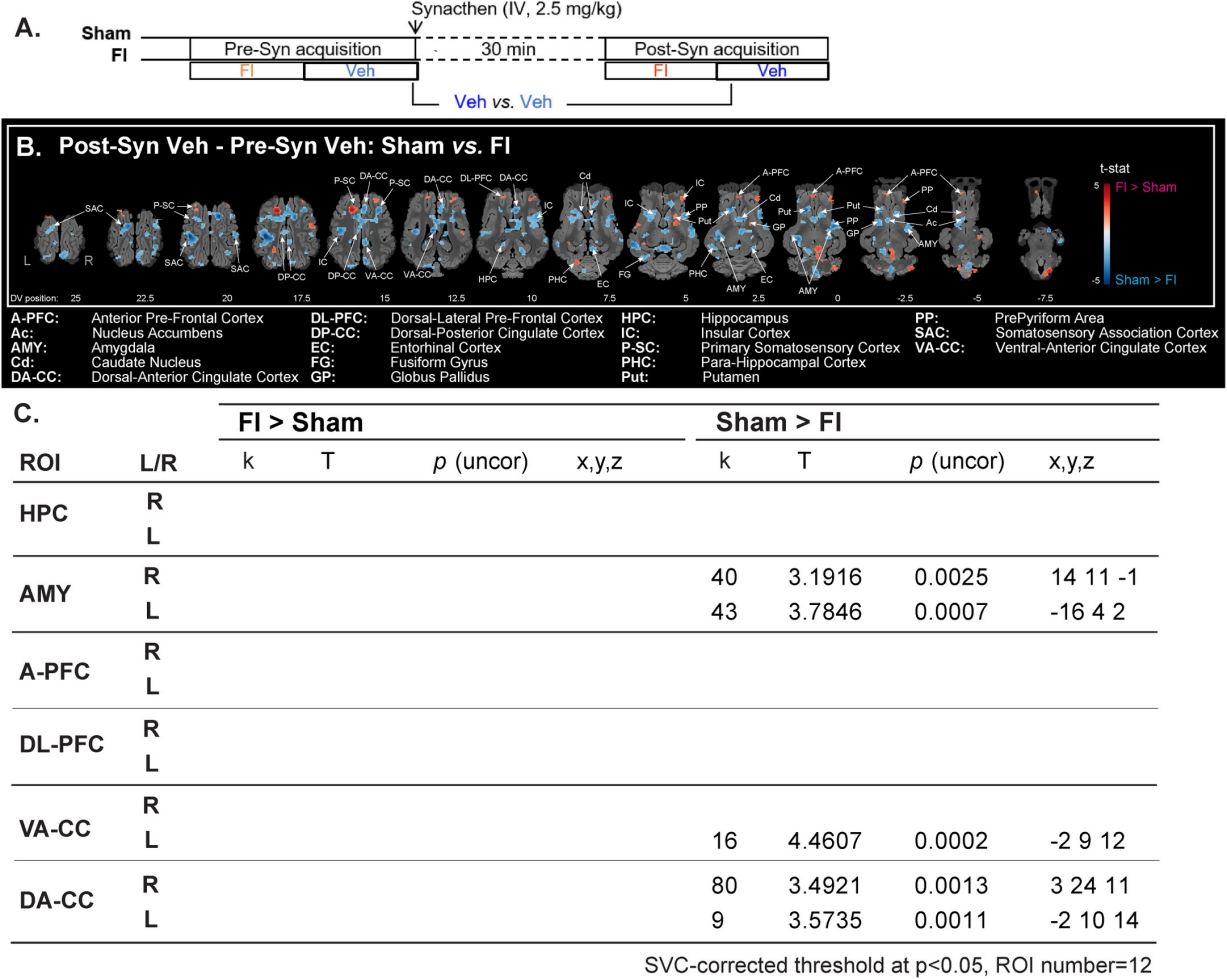
Supplementation with the food ingredient attenuated the global increased brain activity elicited by the pharmacological stress. **(A)** Increased brain activity in response to the injection of pharmacological ACTH agonist (Synacthen®) was compared between supplemented (FI, n = 10) and not supplemented (Sham, n = 10) animals. FI: Olfactory stimulation with the food ingredient, Veh: Neutral stimulation with the vehicle. **(B)** Horizontal maps of brain BOLD increased responses to the injection in both groups. p-value threshold = 0.05, k > 20, DV: Dorsal-ventral position related to the posterior commissure (in mm). The part of the frontal cortices that was not covered with the average BOLD-based statistical maps is superimposed in dark grey on the anatomical maps. **(C)** SVC-based statistics: Related regions of interest (ROIs) with uncorrected p-value that reached the criteria of p < 0.05 after Bonferroni correction.

The imaging contrast used to assess the effect of a previous supplementation of FI on the brain responses to the pharmacological stress is presented in Fig 6A. The pharmacological stress-induced enhanced activity was reduced in FI animals compared with the Sham animals (brain activation maps, Fig 6B*)*. First, the SAC, P-SC, EC, IC, GP and FG were more activated in Sham. To a lesser extent, the P-SC, IC and GP were also more activated in FI animals. The HPC, AMY, PHC and cingulate cortex (DA, DP and VA) were more activated in Sham, as well as the reward/motivational zones (Cd, Put and Ac). At the opposite, the A-PFC was more activated in FI than in Sham animals.

#### Corrected SVC-based statistic (Fig 6C)

The pharmacological stress induced higher bilateral activation in the AMY (right, *p* = 0.0025 and left, *p* = 0.0007), DA-CC (right, *p* = 0.0013 and left, *p* = 0.0011) and in the left VA-CC (*p* = 0.0002) of Sham animals. We could not detect any higher brain activation in FI animals compared with Sham animals.

## Discussion

This study investigated the fMRI BOLD responses to a food ingredient (FI) and an acute pharmacological activation of the HPA axis in the context of a pig chronic stress model. Our results demonstrated that: 1) Chronic supplementation with the FI promoted familiarization and further memory retrieval processes; 2) The pharmacological acute stress induced specific activations in brain regions associated with arousal, associative learning and cognition; and 3) The supplementation with FI alleviated the brain responses to acute stress, which might suggest that FI could help coping with repeated stressful events and eventually reduce chronic stress.

### Supplementation with the food ingredient promoted familiarization processes

Animals were subjected to the chronic stress paradigm previously validated in our research department [33]. We had previously shown that chronic stress housing conditions were notably associated with the onset of behavioural despair, a core symptom of depression, and with HPA axis secretion deregulation. This model had also been successfully used to study the effects of a food ingredient principally made of spices extracts on the microbiota-gut-brain axis and cerebral blood perfusion [34]. Several studies have reported anxiolytic- and/or antidepressant-like effects as well as HPA axis modulation through environmental/oral exposure to *Citrus* extracts [22, 27–29]. Moreover, a study investigating the effects of the same sensory functional food ingredient (D11399) orally administered in a mouse model of mood disorders demonstrated anxiolytic- and antidepressant-like effects associated with a modulation of the serotoninergic system and hippocampal plasticity [40]. In the present study, we could not detect any effects of the FI supplementation on behavioural despair and HPA axis. We hypothesize that either the cottage cheese matrix changed the functional properties of the FI or its detection by the animals, or the dose and/or duration of exposure per day was not sufficient to provide behavioural and physiological outcomes. It is also possible that the stressor procedure, in this study, did not induce sufficient effects on behavioural despair and cortisol levels, thus preventing FI supplementation to exert effects on these parameters, although in a previous study we showed that this procedure was effective [33]. Coutens et al. [40] also showed that the anxiolytic- and antidepressant-like effects of the same FI are driven by an activation of the olfactory system, as a deactivation of olfactory epithelium by the pharmacological agent methimazole suppresses these effects. However, the brain responses to the olfactory stimulation with the FI during the fMRI session differed between groups, suggesting that the concentration of FI used in the food supplementation was sufficient to be detected, learned, and to trigger differential brain activity in comparison to animals that did not receive it, which can be considered as familiarization. Indeed, in FI compared to Sham groups, the stimulation elicited a higher activation in regions involved in the olfactory perception, in associative learning and emotional processing, and in the reward and motivational circuits. The fact that olfactory perception and sensory integration regions were more activated by the ingredient is particularly relevant and suggests that familiarization to the ingredient appeared despite the lack of behavioural and endocrine evidences. Indeed, learning through the repeated exposure to an odour induces connectivity changes in the olfactory bulb [41–44], which might lead to a decreased perception threshold after habituation and a subsequent higher activation of perception centres. Thus, acute olfactory exposure is susceptible to induce more important brain responses in pre-supplemented animals. Particularly, we had previously shown that the same active sensory core as that used in the present FI elicited the activation of regions implicated in reward and motivational regions in animals that had never encountered it [21], and that this activation was increased in pre-supplemented animals [32], which is also the case in this study. This comes together with a higher activation of regions involved in associative learning and emotional processing, which might support this hypothesis. At the opposite, Sham animals showed higher bilateral brain responses in the DA-CC, which might be a sign of an increased arousal due to the presentation of a new odour [45, 46], and thus potentially to neophobia.

All these results suggest that, despite the lack of evidences at the behavioural and HPA axis levels, animals pre-supplemented during the experimental period had neuromodulatory effects, and that an acute stimulation with the ingredient might be more hedonic in animals that had been used to it.

Finally, the observation of ambivalent brain activity within the same anatomical area (*e*.*g*. DA-CC) is not surprising, especially when the brain area is large and involved in different types of processes. Different subdivisions of a given brain area can relate to different processes, sometimes leading to the concomitant observation of activations and deactivations within the same area. The functional segregation of the DA-CC, for example, has been well described [47, 48] but mechanistic studies are lacking in the pig model. Though, it is likely that the DA-CC subdivision that was activated in FI compared to Sham is not involved in the same functional response as that of the subdivision activated in Sham compared to FI. Behavioural correlates only might help interpreting these results, but the behavioural tests used in our study failed in highlighting differences between groups. Other tests should be implemented in this model to disentangle the respective behavioural functions (cognitive control, emotions, memory, *etc*.) of brain areas with ambivalent responses such as DA-CC.

### Pharmacological acute stress increased the brain activity in chronically stressed animals

As an ACTH agonist, Synacthen^®^ has stimulating effects on the activity of the HPA axis and was thus employed as a pharmacological agent to induce an acute stress modelled through an elevation of cortisol level during imaging. In the present study, Synacthen injection promoted an increased brain activity in chronically stressed animals, during the neutral olfactory stimulation with the vehicle, notably in the hippocampus and in regions involved in sensory perception and arousal. It is well documented that glucocorticoids interact with the arousal state [49] and that their signaling, notably in the hippocampus, amygdala and DL-PFC, play a critical role in encoding, processing, and retaining emotional and stressful events [50]. Consolidating such memory is an adaptive response that might be necessary for appropriate reactions to further similar situations [50]. However, pathological conditions such as chronic stress and mood disorders can interact with memory consolidation systems and lead to impaired cognition. We did not investigate the consequences of our chronic stress model on memory yet, but the observed decreased activity in amygdala and absence of modulation of the dlPFC require further studies to challenge the hypothesis of an impaired cognition. Specific behavioural tests aimed at investigating spatial learning and memory, with the use of different types of rewards/reinforcers, might be implemented in the context of this model, such as the holeboard and maze tests for example [51, 52]. Responses in the A-PFC were contrasted as several sub-parts of the A-PFC had a higher level of activation after the injection (left) and several other sub-parts had a lower bilateral activity. However it is possible to argue that, as the A-PFC provides top-down regulation of highly cognitive functions [53], acute stress might inhibit this network in order to enable a quicker behavioural response to the stressful event. An increased activity was also found in the cingulate cortex, and imaging studies combined with the clinical MIST task associated with an increased cortisolemia have also reported an increased activity in this brain region in the human [12].

The pharmacological stress also induced an increased activity in the reward and motivational networks. The links between acute stress and reward/motivational circuits have been investigated in several studies, and the reward system is known to be modulated in different aspects in mood disorders and by acute stress [54]. Especially, acute stress is associated with an increased activity of these regions during reward anticipation after a Pavlovian conditioning [55], but in our context the reason of this increased activity remains unclear.

### Supplementation with the food ingredient attenuated the global pharmacological stress-induced increased brain activity

We found that the pharmacologically-induced enhanced brain activity was lower in FI compared to Sham animals. Particularly, regions associated with perception and arousal as well as hippocampus had a lower activity in FI than in Sham animals. It was also the case for the regions of the cingulate cortex that were more activated after the injection (DA, VA and DP-CC) in FI animals. The cingulate cortex is known to be involved in multiple different functions. In short, the A-CC receives inputs from the OFC and is involved in emotion and reward, whereas the P-CC has outputs to the hippocampal system and is rather involved in memory [47]. Interestingly, recent work showed that patients with post-traumatic stress disorder have an altered resting-state DA-CC functional connectivity [56]. This particular structure is also known to be involved in the cognitive control over decision and action, and notably during foraging behaviour [48, 57]. Our previous imaging studies have shown that this FI was able to stimulate brain regions involved in stress responses, and these modulations were associated with behavioural advantages on the day of a stressful feed transition (*i*.*e*. alleviation of stress-induced anorexia) [32, 58]. No behavioural nor physiological evidence of benefits from the FI in the context of chronic stress conditions was found in the present study, even though the first fMRI acquisition showed that FI animal were successfully familiarized to the ingredient. Thus, whether these attenuated brain responses to acute stress are beneficial is not proved yet, as there is still no behavioural correlate, but this would deserve further investigations, especially with other more discriminative behavioural testing protocols than those already used in this study (*e*.*g*. openfield test for testing behavioural reactivity, holeboard and maze tests for testing spatial cognitive abilities with different types of rewards/reinforcers, *etc*.). Moreover, this FI has also shown beneficial behavioural effects during an acute stress on animals undergoing a chronic stress protocol [40]. The fact that many of the regions modulated by the olfactory stimulation with FI are the same as the regions activated by the acute pharmacological stressor indicates that the strategy of using olfactory FI to modulate the responses to stress is promising and needs further behavioural demonstrations.

### Methodological considerations for further studies

It has to be noticed that the fMRI data need however to be taken with caution because of a potential effect of anaesthesia on the brain responsiveness to the olfactory stimulation. Indeed, anaesthetized animals might respond differently compared with awake animals [59]. Isoflurane anaesthesia has also been described to modulate differently the brain responsiveness in different brain structures [60]. In addition, we could also consider that acute pharmacological stress might alleviate to some extent the depth of isoflurane-based anaesthesia, and thus promote an increased BOLD signal. However, under pharmacological stress, given that we detect higher brain activation in response to olfactory stimulation compared to sham stimulation, we suggest that the BOLD signal increase under pharmacological stress cannot be related to a potential alleviation of anaesthesia control.

The BOLD acquisitions are performed here with a human set-up (magnet and antenna) that might cause inconvenience possibly corrected with smaller dedicated antenna for pig head as example. For instance, the spatial resolution (2.5x2.5x2.5 mm3) of the BOLD acquisition might also not promote sufficient spatial parcellation for a satisfactory covering of small brain region, such as the nucleus accumbens. Another limitation is that BOLD acquisition with echo planar imaging might cause spatial distortions, therefore possibly reducing the confidence in the spatial attribution of a voxel in a specific ROI. This has to be taken into consideration for data interpretation.

Here, we decided to use an fMRI approach with a neutral olfactory stimulation in order to assess the brain responses to a pharmacological stress. For further studies, a resting state fMRI approach should yield additional information regarding the impact of pharmacological stress on brain functional connectivity, as already described in the pig model with nucleus accumbens stimulation [37].

It is also important to notice that females only were included in this study, to maximize the statistical power during brain imaging. Our previous study conducted in male and females had shown that no difference was observed in response to the chronic stressor [33], however the brain responses to acute pharmacological stress and to the FI might be different. Further imaging studies might thus also include both sexes.

Finally in this study, we did not manage to show any significant behavioural effect of the FI during the restraint test. This could mitigate the positive effects observed at the brain level, but we must remind that the restraint test was adapted from high-stress standardized tests in rodents, which were developed to assess depression symptoms such as behavioural despair. The absence of difference for this test in our model does not predict what the behavioural responses of our animals would be in other and perhaps less constraining testing conditions. To further explore this hypothesis, additional behavioural tests could be performed to investigate cognitive and emotional abilities (e.g. behavioural reactivity to novelty, learning and memory tests) or perseverance in different situations involving goal-oriented behaviours and motivation (e.g. operant conditioning or maze tests rewarded by resources or access to social partners). Further studies could for example include the novel object test, that has been adapted in pigs to study reactivity to novelty and learning abilities [61, 62], or the holeboard test, in which animals have to learn the position of food reward [52, 63].

## Conclusions and perspectives

First, this study shows that it is possible to observe brain responses to a pharmacological stress using fMRI. In the pig psychosocial chronic stress model that we used, these responses consist in a global increase of brain activity in brain regions related to arousal, perception, emotional processing and associative learning. Further studies would be necessary to investigate the effects of acute stress on brain responses in pigs that are not subjected to chronic stress. Secondly, we suggested potential beneficial effects of a supplementation with the sensory functional food ingredient D11399, mainly made of *Citrus sinensis* extracts, on the brain responses to acute stress. Indeed, the FI supplementation reduced the acute stress-induced increased brain activity in chronically stressed animals. As an overstimulation of these brain networks by repeated acute stress might be a cause of mood disorders, prolonged oral supplementation with this FI might be a way to prevent the onset of supplementary neurophysiological and behavioural disturbances associated with acute stress in a mood disorder context.
